# AGE-related efficacy and safety of darolutamide triplet therapy in metastatic hormone-sensitive prostate cancer: are we ready for routine clinical practice?

**DOI:** 10.3389/fonc.2026.1971526

**Published:** 2026-10-05

**Authors:** Fernando Munoz, Francesco Fiorica

**Affiliations:** 1 Department Radiation Oncology, Aosta, Italy; 2 Department of Medical Oncology, Aulss 9 Scaligera, Verona, Italy

**Keywords:** age patients, darolutamide, frailty, metastatic hormone-sensitive prostate cancer, treatment intensification

## Introduction

The treatment of metastatic hormone-sensitive prostate cancer (mHSPC) has rapidly evolved, with treatment intensification becoming an established strategy. The ARASENS trial demonstrated a survival benefit from adding darolutamide to androgen deprivation therapy (ADT) and docetaxel. However, the optimal management of older patients remains challenging, as this population is often underrepresented in clinical trials and chronological age does not necessarily reflect biological fitness or treatment tolerance.

The recent age-based analysis by Carles and colleagues provides important information on the efficacy and safety of darolutamide triplet therapy across age groups. Nevertheless, several methodological and clinical issues should be considered before these findings are broadly applied to older patients in routine practice. In this Opinion article, we discuss these limitations and the need for a more individualized, geriatric-informed approach to treatment selection in older patients with mHSPC.

## Discussion

We read with great interest the article by Carles and colleagues evaluating the efficacy and safety of darolutamide in combination with androgen deprivation therapy (ADT) and docetaxel according to patient age in metastatic hormone-sensitive prostate cancer (mHSPC). The authors should be congratulated for addressing one of the most relevant challenges in contemporary uro-oncology, namely the optimization of treatment strategies for older patients, a population that is rapidly increasing in daily clinical practice but remains underrepresented in prospective clinical trials.

The age-based analysis of the ARASENS trial suggests that the addition of darolutamide to ADT plus docetaxel provides consistent survival benefits with a manageable safety profile irrespective of age. Nevertheless, we believe that several methodological and clinical aspects deserve further consideration before these findings are broadly translated into routine practice.

First, the analysis was exploratory and conducted *post hoc*. Because the study was not prospectively designed or statistically powered to evaluate treatment effects according to age, the apparent consistency observed across age subgroups should be interpreted cautiously. As with all *post hoc* subgroup analyses, the possibility of insufficient statistical power and chance findings cannot be excluded, and these results should therefore be regarded as hypothesis-generating rather than conclusive (1, 2).

Second, the population aged ≥75 years enrolled in ARASENS represents a highly selected subset of older adults. Eligibility criteria required an ECOG performance status of 0–1 and adequate organ function to receive docetaxel, effectively excluding many elderly patients commonly encountered in clinical practice who present with frailty, multimorbidity, polypharmacy, or reduced physiological reserve. Consequently, the generalizability of these findings to the real-world geriatric population remains uncertain (3, 4).

An additional limitation is the use of chronological age as the principal stratification variable. Increasing evidence indicates that chronological age alone is a poor surrogate for biological fitness and treatment tolerance. Neither a comprehensive geriatric assessment (CGA) nor validated frailty screening instruments were incorporated into the study protocol, despite recommendations from the International Society of Geriatric Oncology (SIOG) and other expert societies advocating treatment decisions based on functional rather than chronological age (4, 5).

Furthermore, although treatment-emergent adverse events were generally comparable between age groups, the study did not investigate outcomes that are particularly meaningful in elderly patients, including preservation of functional independence, cognitive function, mobility, falls, or patient-reported quality of life. These clinically relevant domains are often insufficiently reflected by conventional CTCAE toxicity reporting yet frequently influence therapeutic decision-making in older adults (5).

Finally, the therapeutic paradigm for mHSPC has evolved considerably since the conception of ARASENS. Although the trial firmly established the benefit of darolutamide combined with ADT and docetaxel, it does not answer the increasingly important question of whether fit older patients derive incremental benefit from triplet therapy compared with contemporary doublet regimens consisting of ADT plus an androgen receptor pathway inhibitor alone. In current practice, treatment decisions in elderly patients should therefore remain individualized, integrating disease burden, life expectancy, comorbidity, frailty, patient preferences, and therapeutic goals, in accordance with current SIOG recommendations (4).

Looking ahead, future clinical research should specifically focus on the older population through prospective studies adequately powered to evaluate treatment efficacy according to both chronological age and biological fitness. Incorporation of CGA and validated frailty screening tools, such as the G8 questionnaire or the Clinical Frailty Scale, should become a routine component of study design to improve patient selection for treatment intensification (3, 5).

Equally important, future trials should include geriatric-specific endpoints—including maintenance of functional autonomy, cognitive outcomes, health-related quality of life, treatment burden, and patient-reported outcomes—alongside conventional oncological efficacy measures. Randomized studies directly comparing triplet therapy with modern doublet strategies in biologically fit older adults are also needed, together with investigations evaluating treatment de-escalation approaches for frail patients and predictive biomarkers capable of identifying individuals most likely to benefit from intensified systemic therapy. Finally, prospective real-world registries enrolling unselected elderly patients will be essential to confirm the external validity of clinical trial results and to support more individualized therapeutic decision-making in everyday clinical practice.
